# Sitagliptin may reduce prostate cancer risk in male patients with type 2 diabetes

**DOI:** 10.18632/oncotarget.12137

**Published:** 2016-09-20

**Authors:** Chin-Hsiao Tseng

**Affiliations:** ^1^ Department of Internal Medicine, National Taiwan University College of Medicine, Taipei, Taiwan; ^2^ Division of Endocrinology and Metabolism, Department of Internal Medicine, National Taiwan University Hospital, Taipei, Taiwan; ^3^ Division of Environmental Health and Occupational Medicine of the National Health Research Institutes, Zhunan, Taiwan

**Keywords:** incretin, National Health Insurance, prostate cancer, sitagliptin, Taiwan

## Abstract

This retrospective cohort study evaluated the risk of prostate cancer associated with sitagliptin use in Taiwanese male patients with type 2 diabetes mellitus by using the reimbursement databases of the National Health Insurance. Male patients with newly diagnosed type 2 diabetes mellitus at an age ≥25 years between 1999 and 2010 were recruited. A total of 37,924 ever users of sitagliptin and 426,276 never users were followed until December 31, 2011. The treatment effect of sitagliptin (for ever versus never users, and for tertiles of cumulative duration of therapy) was estimated by Cox regression incorporated with the inverse probability of treatment weighting using propensity score. Analyses were also conducted in a 1:1 matched pair cohort based on 8 digits of propensity score. Results showed that during follow-up, 84 ever users and 2,549 never users were diagnosed of prostate cancer, representing an incidence of 140.74 and 240.17 per 100,000 person-years, respectively. The hazard ratio (95% confidence intervals) for ever users versus never users was 0.613 (0.493-0.763). The respective hazard ratio for the first, second, and third tertile of cumulative duration of sitagliptin use <5.9, 5.9-12.7 and >12.7 months was 0.853 (0.601-1.210), 0.840 (0.598-1.179) and 0.304 (0.191-0.483), respectively; and was 0.856 (0.603-1.214), 0.695 (0.475-1.016) and 0.410 (0.277-0.608) for cumulative dose <15,000, 15,000-33,600 and >33,600 mg, respectively. Findings were supported by analyses in the matched cohort. In conclusion, sitagliptin significantly reduces the risk of prostate cancer, especially when the cumulative duration is >12.7 months or the cumulative dose >33,600 mg.

## INTRODUCTION

The incidence of prostate cancer varies among different countries by 25-fold with higher incidence in developed countries in Europe and North America and lower incidence in less developed countries in Asia and Africa [1]. While the temporal trends of prostate cancer incidence are decreasing in developed countries, they are increasing remarkably in the Asian populations [1]. In Taiwan, the age-standardized incidence of [2] and mortality from [3] prostate cancer are both increasing.

Patients with type 2 diabetes mellitus (T2DM) suffer from a higher risk of cancer, which can be related to obesity, insulin resistance, hyperinsulinemia, glycemic control, comorbidities or antidiabetic drugs [4–11]. Prostate cancer is probably the only cancer that shows a reduced risk in patients with T2DM in studies conducted mainly in the white people [12, 13]. However, studies conducted in Taiwan suggested a significantly higher risk of prostate cancer in patients with T2DM in terms of incidence [2], prevalence [14] and mortality [3]. A recent meta-analysis conducted in the Asian populations also strongly supported a higher risk of prostate cancer in patients with T2DM [15]. The use of antidiabetic drugs may affect the risk of prostate cancer. For example, metformin significantly reduces prostate cancer risk [16], but insulin was neither predictive for its incidence [17] nor for its mortality [18].

Incretin-based therapies by using dipeptidyl peptidase-4 (DPP-4) inhibitors or glucagon-like peptide-1 receptor (GLP-1R) agonists have become a mainstay in the treatment of T2DM. However, the cancer risk associated with these drugs remains to be clarified [19]. Sitagliptin was the first DPP-4 inhibitor approved for clinical use in 2006 and it was approved for reimbursement by the Taiwan's Bureau of National Health Insurance (NHI) on March 1, 2009 [19]. Sitagliptin is probably the most commonly used incretin-based therapy but its association with prostate cancer has not been extensively explored. A pooled analysis of 25 clinical studies including 7,726 users and 6,885 non-sitagliptin treated patients with study durations of 12 weeks to 2 years showed that the incidence of prostate cancer was 0.11 and 0.07 per 100 patient-years, respectively [20]. However, the incidence for metastatic prostate cancer or stage III prostate cancer were both lower in the sitagliptin group (0.00 vs. 0.02 per 100 patient-years) [20]. Because of the small numbers of incident cancer, statistical analyses were not performed in the study.

The present study aimed at evaluating whether sitagliptin use in male patients with T2DM would affect the risk of prostate cancer by using the Taiwan's NHI reimbursement database. Other incretins (i.e., saxagliptin, vildagliptin and linagliptin for DPP-4 inhibitors; and exenatide and liraglutide for GLP-1R agonists) currently available in Taiwan were not evaluated because they were not approved until after 2009 and had not been used commonly during the study period. A new-user design was applied to minimize the potential “prevalent user bias” [21]. To reduce the “immortal time bias” (the initial period of follow-up during which the outcome can not occur) [22], patients should have been prescribed antidiabetic drugs for at least two times, and those who were followed up for a short period of time (i.e., <180 days) were excluded. To address the imbalance in characteristics associated with treatment allocation in non-random observational studies, Cox regression models were created by incorporation with the inverse probability of treatment weighting (IPTW) using propensity score (PS) [23] and analyses were also conducted in a 1:1 matched cohort [24].

## RESULTS

Table 1 compares the characteristics between ever and never users of sitagliptin in the original cohort and the matched cohort, respectively. In the original cohort, except for stroke, dipyridamole and examination of prostate-specific antigen (PSA), all variables differed significantly. Ever users were younger in age, had longer duration of diabetes, and characterized by higher proportions of hypertension, nephropathy, ischemic heart disease, peripheral arterial disease, eye disease, obesity, dyslipidemia and acute pancreatitis. On the other hand, lower proportions of chronic obstructive pulmonary disease, and benign prostatic hyperplasia were observed in ever users. Ever users were more likely to use other medications. Among the covariates, 19 of the 28 had values of standardized difference >10%. In the matched cohort, only diabetes duration, and use of metformin and rosiglitazone were different significantly. However, while examining the standardized differences, none of them had a value >10%.

**Table 1:** Comparison of characteristics between sitagliptin never and ever users in the original cohort and the matched cohort, respectively

Variables	Original cohort						Matched cohort					
	Sitagliptin				*P* value	SD	Sitagliptin				*P* value	SD
	Never users		Ever users				Never users		Ever users			
	n	%	n	%			n	%	n	%		
	426276		37924				37792		37792			
Age* (years)	54.23	12.92	51.49	11.49	<0.0001	−12.37	51.61	11.50	51.51	11.49	0.2263	−1.05
Diabetes duration* (years)	4.36	3.29	6.25	3.29	<0.0001	24.14	6.14	2.98	6.24	3.29	<0.0001	2.80
Hypertension	313104	73.45	30095	79.36	<0.0001	15.83	29935	79.21	29984	79.34	0.6601	0.16
COPD	206970	48.55	16663	43.94	<0.0001	−19.46	16457	43.55	16618	43.97	0.2378	0.81
Stroke	116894	27.42	10319	27.21	0.3741	−6.23	10281	27.20	10280	27.20	0.9935	−0.08
Nephropathy	108153	25.37	10086	26.60	<0.0001	−2.91	9999	26.46	10059	26.62	0.6211	0.26
Ischemic heart disease	173371	40.67	16590	43.75	<0.0001	−1.63	16314	43.17	16508	43.68	0.1545	0.93
Peripheral arterial disease	80906	18.98	8213	21.66	<0.0001	1.76	8163	21.60	8185	21.66	0.8459	0.10
Eye disease	55643	13.05	11466	30.23	<0.0001	48.12	11492	30.41	11377	30.10	0.3625	−0.72
Obesity	14747	3.46	2050	5.41	<0.0001	10.15	1974	5.22	2026	5.36	0.3982	0.62
Dyslipidemia	304825	71.51	31233	82.36	<0.0001	25.27	31030	82.11	31110	82.32	0.4467	0.48
Acute pancreatitis	8758	2.05	940	2.48	<0.0001	−0.22	904	2.39	936	2.48	0.4501	0.56
BPH	152951	35.88	12034	31.73	<0.0001	−14.16	12083	31.97	12009	31.78	0.5635	−0.47
Statin	198784	46.63	26274	69.28	<0.0001	57.83	26082	69.01	26145	69.18	0.6200	0.21
Fibrate	136135	31.94	16872	44.49	<0.0001	34.02	16629	44.00	16792	44.43	0.2326	0.77
ACEI/ARB	256351	60.14	28041	73.94	<0.0001	36.20	27895	73.81	27914	73.86	0.8751	−0.06
Calcium channel blocker	215115	50.46	20603	54.33	<0.0001	8.94	20416	54.02	20518	54.29	0.4565	0.44
Sulfonylurea	245724	57.64	34906	92.04	<0.0001	120.00	34926	92.42	34774	92.01	0.0391	−1.85
Metformin	266158	62.44	36615	96.55	<0.0001	140.88	36719	97.16	36483	96.54	<0.0001	−3.69
Insulin	52413	12.30	11676	30.79	<0.0001	50.21	11655	30.84	11570	30.61	0.5028	−0.45
Acarbose	67324	15.79	17572	46.33	<0.0001	63.43	17527	46.38	17441	46.15	0.5304	−0.63
Pioglitazone	48007	11.26	15071	39.74	<0.0001	56.45	14918	39.47	14941	39.53	0.8641	0.05
Rosiglitazone	27902	6.55	10939	28.84	<0.0001	44.40	10541	27.89	10809	28.60	0.0304	1.44
Aspirin	223344	52.39	23075	60.85	<0.0001	13.41	22878	60.54	22964	60.76	0.5220	0.32
Ticlopidine	19177	4.50	1921	5.07	<0.0001	0.67	1970	5.21	1905	5.04	0.2837	−0.82
Clopidogrel	36830	8.64	5297	13.97	<0.0001	16.37	5227	13.83	5232	13.84	0.9580	0.00
Dipyridamole	139326	32.68	12517	33.01	0.2016	−2.04	12371	32.73	12485	33.04	0.3774	0.52
Prostate-specific antigen	63539	14.91	5526	14.57	0.0795	0.33	5472	14.48	5497	14.55	0.7963	0.16

^*^Age and diabetes duration are expressed as mean and standard deviation

COPD: chronic obstructive pulmonary disease, BPH: benign prostatic hyperplasia, ACEI/ARB: angiotensin converting enzyme inhibitor/angiotensin receptor blocker, SD: standardized difference

Table 2 shows the incidences and hazard ratios of prostate cancer with regards to sitagliptin exposure. During follow-up, 84 ever users and 2549 never users in the original cohort developed prostate cancer, with respective incidence of 140.74 and 240.17 per 100,000 person-years, and an overall hazard ratio of 0.613 (95% confidence interval: 0.493-0.763). Although the hazard ratios were not significant for the first and second tertiles of cumulative duration and cumulative dose, the risk was significantly reduced in the third tertiles. Analyses conducted in the matched cohort showed similar results.

**Table 2:** Sitagliptin and incidence of prostate cancer and hazard ratios comparing exposed to unexposed

Sitagliptin use	Cases followed	Incident cases of prostate cancer	Person-years	Incidence rate (per 100,000 person-years)	HR	95% CI	*P* value
**I. Original cohort**							
Never users	426276	2549	1061318.16	240.17	1.000		
Ever users	37924	84	59682.53	140.74	0.613	(0.493-0.763)	<0.0001
**Cumulative duration (months)**							
Never users	426276	2549	1061318.16	240.17	1.000		
<5.9	12471	32	16380.28	195.36	0.853	(0.601-1.210)	0.3728
5.9-12.7	12565	34	17697.14	192.12	0.840	(0.598-1.179)	0.3136
>12.7	12888	18	25605.11	70.30	0.304	(0.191-0.483)	<0.0001
**Cumulative dose (mg)**							
Never users	426276	2549	1061318.16	240.17	1.000		
<15,000	12493	32	16340.74	195.83	0.856	(0.603-1.214)	0.3820
15,000-33,600	11927	27	16987.84	158.94	0.695	(0.475-1.016)	0.0602
>33,600	13504	25	26353.96	94.86	0.410	(0.277-0.608)	<0.0001
**II. Matched cohort**							
Never users	37792	174	95836.67	181.56	1.000		
Ever users	37792	84	59473.09	141.24	0.793	(0.607-1.037)	0.0898
**Cumulative duration (months)**							
Never users	37792	174	95836.67	181.56	1.000		
<5.9	12427	32	16324.61	196.02	1.083	(0.735-1.595)	0.6876
5.9-12.7	12524	34	17637.17	192.77	1.068	(0.733-1.556)	0.7338
>12.7	12841	18	25511.31	70.56	0.383	(0.235-0.623)	0.0001
**Cumulative dose (mg)**							
Never users	37792	174	95836.67	181.56	1.000		
<15,000	12453	32	16288.73	196.45	1.093	(0.742-1.611)	0.6524
15,000-33,600	11884	27	16929.47	159.49	0.867	(0.573-1.311)	0.4977
>33,600	13455	25	26254.88	95.22	0.517	(0.339-0.788)	0.0022

Cox regression models were created by incorporation with the inverse probability of treatment weighting using propensity score created from variables in Table 1 HR: hazard ratio, CI: confidence interval

Table 3 shows the overall hazard ratios comparing ever to never users of sitagliptin in sensitivity analyses after excluding patients with various clinical conditions in the original cohort. A significantly reduced risk was observed in all analyses.

**Table 3:** Sensitivity analyses estimating hazard ratios for prostate cancer for ever versus never users of sitagliptin in the original cohort

Model	HR	95% CI	*P* value
I. Excluding cumulative duration of sitagliptin use <180 days	0.613	(0.493-0.763)	<0.0001
II. Excluding patients who had been screened by prostate-specific antigen before prostate cancer diagnosis	0.353	(0.218-0.571)	<0.0001
III. Excluding patients with a diagnosis of other cancers during follow-up	0.612	(0.492-0.761)	<0.0001
IV. Excluding patients with a diagnosis of acute pancreatitis during follow-up	0.623	(0.501-0.775)	<0.0001
V. Excluding patients with a diagnosis of acute pancreatitis and/or other cancers during follow-up	0.621	(0.499-0.773)	<0.0001
VI. Excluding patients aged <45 years	0.683	(0.549-0.849)	0.0006

HR: hazard ratio, CI: confidence interval

## DISCUSSION

The results support an overall reduced risk of prostate cancer in sitagliptin users. The reduced risk is significantly observed in patients treated with sitagliptin for more than one year or with a cumulative dose >33,600 mg (Table 2). The matched cohort analyses (Table 2) and the sensitivity analyses (Table 3) supported the finding.

It is interesting that such a reduced risk of prostate cancer related to incretin-based therapies is also observed in a recent clinical trial on liraglutide, a GLP-1R agonist, showing a hazard ratio (95% confidence interval) of 0.54 (0.34-0.88) [25]. Though with a slightly smaller magnitude of risk reduction, the overall hazard ratio (95% confidence interval) of 0.613 (0.493-0.763) estimated for sitagliptin in the present study (Table 2) was very close to the observed hazard ratio for liraglutide in the clinical trial [25].

Recent studies from Japan may provide some explanations for the potential mechanisms. The Japanese investigators showed that GLP-1R is expressed in human prostate cancer tissue and exendin-4 (a GLP-1R agonist) significantly decreased the proliferation of prostate cancer cells that expressed GLP-1R [26, 27]. Such antiproliferative effect of exendin-4 on prostate cancer was not via androgen receptor, but through the inhibition of extracellular signal–regulated kinase-mitogen-activated protein kinase (ERK-MAPK) phosphorylation via GLP-1R [26]. This inhibitory effect on ERK-MAPK was mediated by the cyclic adenosine 3′,5′-monophosphate-protein kinase A pathway and was not dependent on adenosine monophosphate activated protein kinase activation or Akt phosphorylation [26]. In an *in vivo* study, exendin-4 also attenuated the growth of prostate cancer cells transplanted to athymic mice via the inhibition of ERK-MAPK activation [26]. In humans, administration of sitagliptin 50 mg per day for 3 days significantly increased active GLP-1 level by 2.5–fold [28]. Therefore, sitagliptin may exert an anti-cancer effect on the prostate by increasing the acitve GLP-1 levels.

On the other hand, DPP-4 may inhibit the malignant phenotype of prostate cancer cells via the blockade of the signaling pathway of basic fibroblast growth factor [29] and the serum activity of DPP-4 is reduced in patients with metastatic prostate cancer [30]. Therefore, inhibition of DPP-4 may potentially trigger prostate cancer cell growth and metastasis, as shown in an *in vitro* and *in vivo* metastasis study [31]. The dual effects of DPP-4 inhibition by sitagliptin may explain the slightly smaller magnitude of risk reduction for sitagliptin (Table 2) than that observed in the liraglutide trial [25].

Our previous analyses of the same database suggested that sitagliptin may increase the risk of acute pancreatitis [32], pancreatic cancer [33] and thyroid cancer [34]. Competing risk due to the development of other cancers and/or acute pancreatitis related to sitagliptin exposure during follow-up would not affect the finding (Table 3). Furthermore, detection bias due to PSA screening could not explain the reduced risk related to sitagliptin because the examination of PSA did not differ significantly between ever and never users (Table 1) and the finding was not changed after excluding patients who had been screened by PSA (Table 3).

There are several strengths in this study. First, a special request was made to include longitudinal data of all patients, covering a period from 1996 when the NHI database was available. Second, the study enrolled a large sample size representing the whole nation. Third, information bias related to self-reporting could be reduced by the use of medical records. Fourth, because the NHI covers almost the entire population, the results could be readily generalized to the whole population.

There are some limitations. First, we did not have biochemical data such as blood levels of glucose and lipid profiles for evaluating their impacts. Second, this study could not evaluate the effects of other DPP-4 inhibitors and GLP-1R agonists because of the limited sample sizes of users of these medications during the study period. Third, it was not possible to fully adjust for potential confounders such as obesity, smoking, alcohol drinking, family history, life-style, dietary factors, hormonal profiles and genetic parameters.

In summary, this study supports an overall reduced risk of prostate cancer in male patients with T2DM who have been treated with sitagliptin. The risk is significantly reduced when the cumulative duration is >12.7 months or the cumulative dose >33,600 mg.

## MATERIALS AND METHODS

This study was approved by an ethic review board of the National Health Research Institutes (NHRI, approval number 99274). The compulsory and universal health care system of NHI has been implemented since March 1995. It covers more than 99% of the Taiwanese population and has contracts with over 98% of the hospitals nationwide. Detailed records including outpatient visits, emergency department visits, and hospital admission are kept in the database, and the information of principal and secondary diagnostic codes, prescription orders, and claimed expenses can be retrieved. According to local regulations, written informed consent was not required because the identification information had already been scrambled prior to the release of the database.

**Figure 1:**
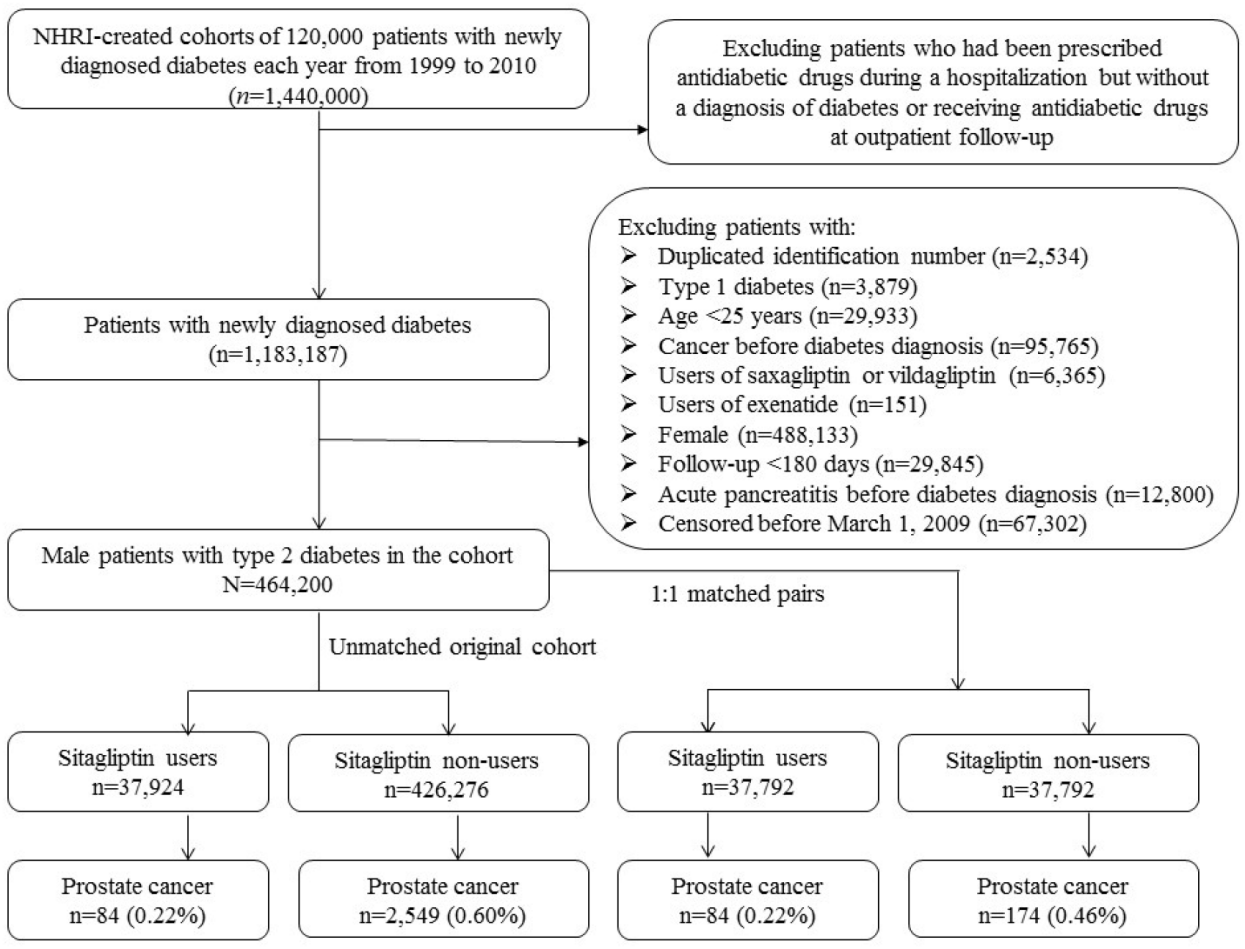
Flowchart showing the procedures followed in creating the unmatched original cohort of male patients with type 2 diabetes mellitus and the 1:1 matched pairs used in the study NHRI: National Health Research Institutes, NHI: National Health Insurance.

Figure 1 shows the procedures followed in creating the cohort of newly diagnosed T2DM patients with onset age ≥25 years for the study. The NHRI created a cohort of 120,000 newly diagnosed diabetic patients in each calendar year for a 12-year period from 1999 to 2010. The longitudinal reimbursement records of these patients from 1996 to 2011 can be provided for academic research after approval. A patient should not have a diagnosis of diabetes in the preceding years when he/she was randomly selected into the cohort for each specific year. The definition of diabetes was based on one of the following two criteria: 1) Diagnosis of diabetes during an admission to the hospital or having been prescribed with antidiabetic drugs during hospitalization; or 2) In an outpatient setting within one year, a patient has been diagnosed as having diabetes for two or more times, or diagnosed as having diabetes for one time plus prescribed with antidiabetic drugs for one time. As a result, a total of 1,440,000 patients with newly diagnosed diabetes were available within these 12 years.

Some patients might have been given insulin or oral antidiabetic drugs temporarily during hospitalization for some medical conditions but they might not be real cases of diabetes. Therefore, patients who were recruited in the yearly established cohorts based on the first criterion but did not have records of follow-up afterward with a diagnosis of diabetes or having been prescribed antidiabetic drugs at the outpatient clinics were excluded. This resulted in a sample size of 1,183,187 patients.

After further exclusion of ineligible patients as shown in the figure (linagliptin and liraglutide were not available in Taiwan during the study period), there were 464,200 patients left. Among them, 37,924 had been prescribed sitagliptin (ever users) and 426,276 had never been treated with sitagliptin (never users).

The *International Classification of Diseases, Ninth Revision, Clinical Modification* (ICD-9-CM) has been used during the study period and diabetes was coded 250.XX, and prostate cancer 185. A number of comorbidities and covariates were determined as a status/diagnosis at the time of censor. The ICD-9-CM codes for the comorbidities were: hypertension 401-405, chronic obstructive pulmonary disease (a surrogate for smoking) 490-496, stroke 430-438, nephropathy 580-589, ischemic heart disease 410-414, peripheral arterial disease 250.7, 785.4, 443.81 and 440-448, eye disease 250.5, 362.0, 369, 366.41 and 365.44, obesity 278, dyslipidemia 272.0-272.4, acute pancreatitis 577.0, and benign prostatic hyperplasia 600. Medications included sulfonylurea, metformin, insulin, acarbose, pioglitazone, rosiglitazone, statin, fibrate, angiotensin-converting enzyme inhibitor and/or angiotensin receptor blocker, calcium channel blocker, aspirin, ticlopidine, clopidogrel, and dipyridamole. Examination of PSA was also considered.

The above characteristics of sitagliptin never users and ever users were compared by Student's t test for age and diabetes duration and by Chi-square test for the others. The crude incidence density of prostate cancer was calculated for sitagliptin ever users and never users and for the tertiles of cumulative duration (months) and cumulative dose (mg). The numerator for the incidence was the number of patients with incident prostate cancer during follow-up, and the denominator was the person-years of follow-up. Follow-up ended on December 31, 2011, at the time of a new diagnosis of prostate cancer, or on the date of the last reimbursement record.

Logistic regression was used to create PS from all the covariate listed in Table 1. To obtained unbiased estimates, Cox regression models were created by incorporation with IPTW using PS as recommended by Austin [23].

To avoid the imbalance of characteristics associated with treatment allocation in non-random observational studies, a 1:1 matched-pair sample based on 8 digits of PS was also used for analyses, with calculation of standardized difference for each characteristic. A standardized difference >10% suggests a potential residual confounding. These methods have been adopted and described in detail in our previous studies [34–37].

The following sensitivity analyses were conducted to estimate the hazard ratios for ever versus never users of sitagliptin in the original cohort by excluding: 1) cumulative duration of sitagliptin use <180 days; 2) patients who had been screened by PSA before prostate cancer diagnosis; 3) patients with a diagnosis of other cancers during follow-up; 4) patients with a diagnosis of acute pancreatitis during follow-up; 5) patients with a diagnosis of acute pancreatitis and/or other cancers during follow-up; and 6) patients aged <45 years.

Analyses were conducted using SAS statistical software, version 9.3 (SAS Institute, Cary, NC). *P*<0.05 was considered statistically significant.
